# Supplementary material for: Effect of non-invasive spinal cord stimulation in unmedicated adults with major depressive disorder: a pilot randomized controlled trial and induced current flow pattern
Source: Mol Psychiatry. 2023 Dec 20;29(3):580–9. doi: 10.1038/s41380-023-02349-9 (PMC11153138; doi:10.1038/s41380-023-02349-9)
Supplement: Supplementary file 1 — Supplementary Information [file 41380_2023_2349_MOESM1_ESM.docx]

**Supplementary Materials**

**Supplementary Figure 1.** CONSORT Flowchart.


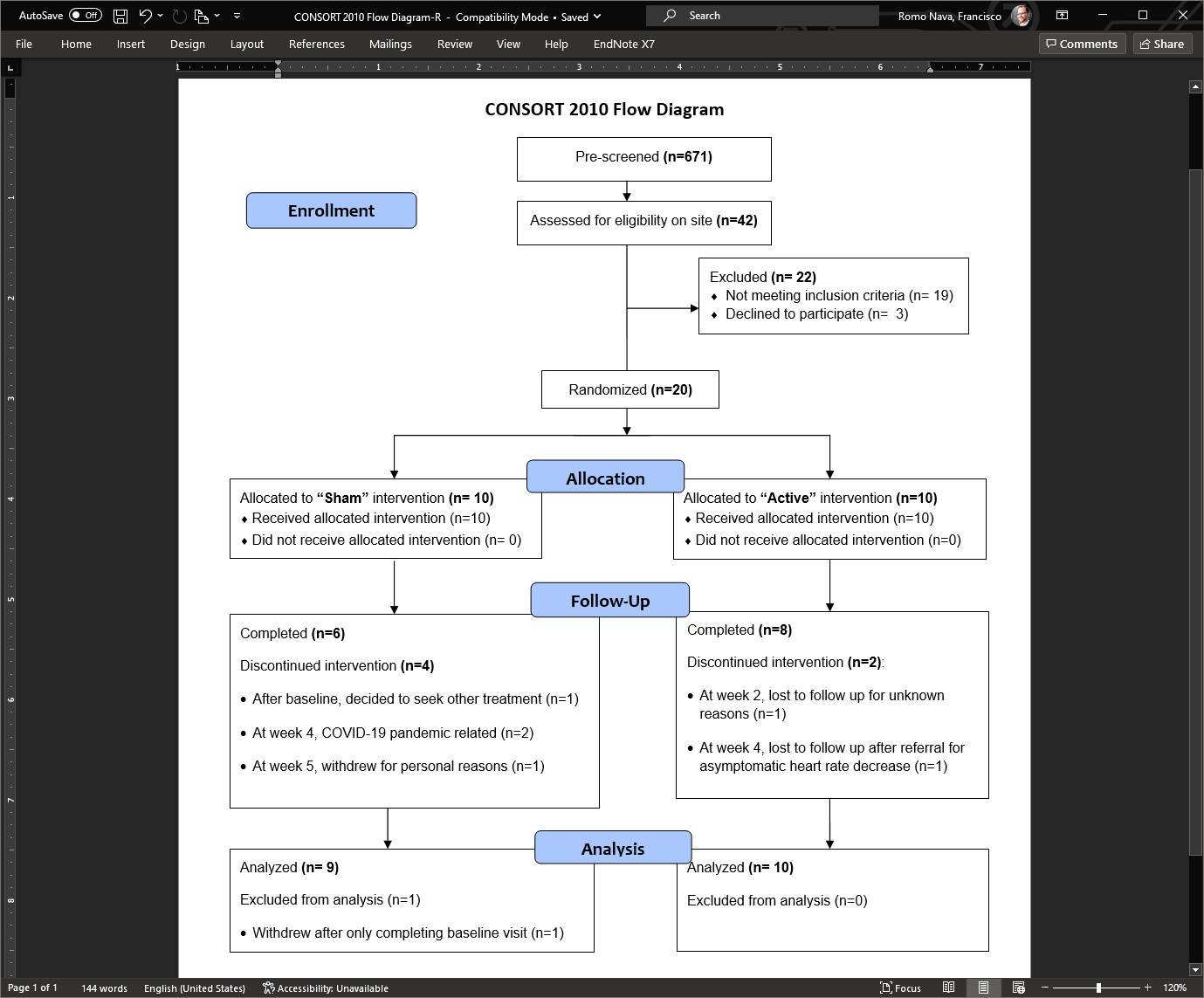


**Supplementary Figure 2.** Correlation between MADRS change and pre/post session systolic blood pressure change from baseline to endpoint (all participants).


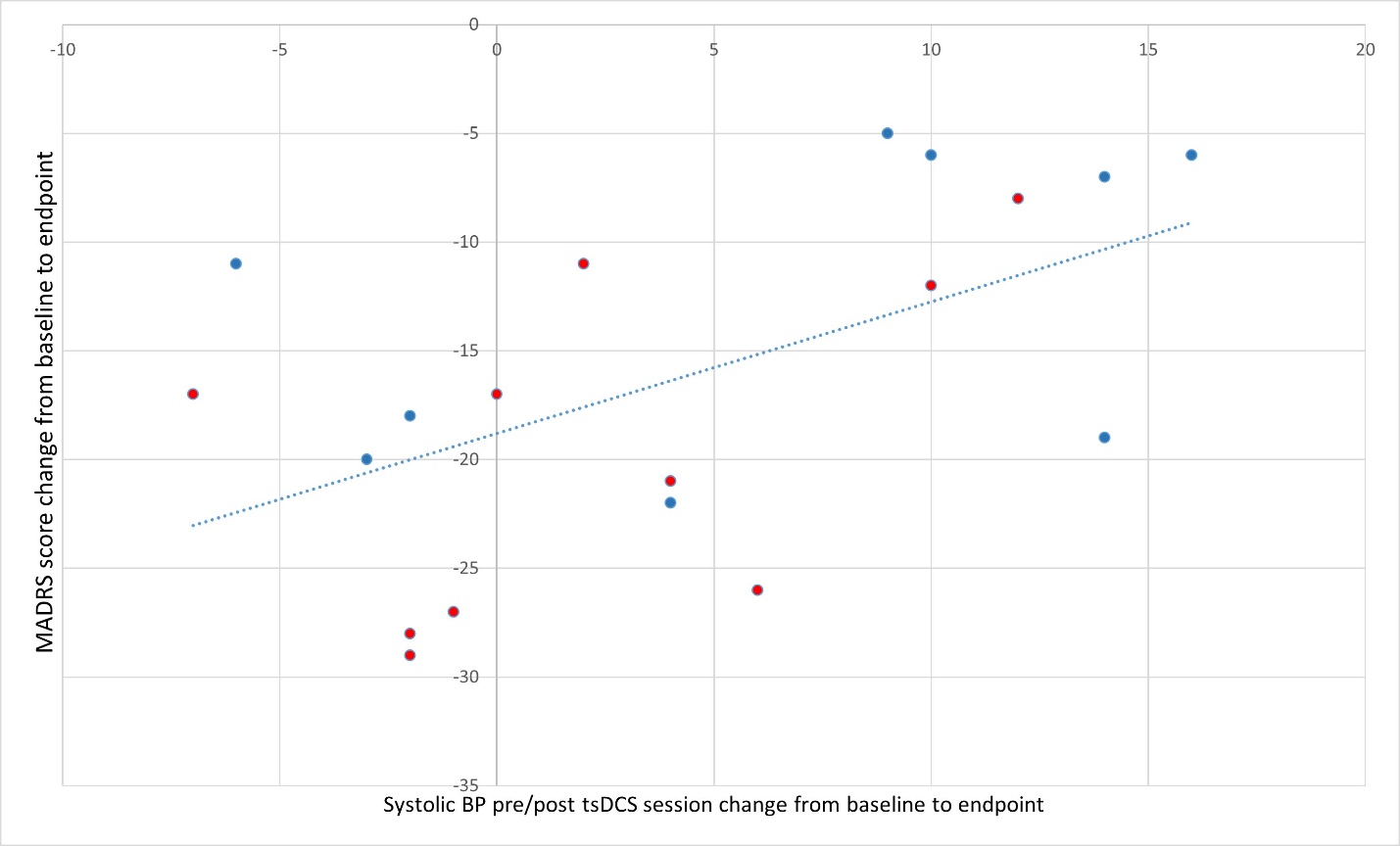


Data points correspond to active (red) or sham (blue) tsDCS groups. Abbreviations: Montgomery Asberg Depression Rating Scale (MADRS); Blood pressure (BP); transcutaneous spinal direct current stimulation (tsDCS).

| **Supplementary Table 1**. Description of the 3D-anatomical dataset that corresponds to the Duke human model from the Virtual Population (ViP)2.0 model. | |
| --- | --- |
| Sex | male |
| Type | Young adult |
| Height (m) | 1.77 |
| Weight (kg) | 70.3 |
| BMI (kg/m^2^) | 22.4 |
| Resolution (mm) | 1 x 1 x 1 |
| Dimension (mm) | 545 x 298 x 1815 |

**Supplementary Table 2.** Categorical response according to MADRS change from baseline to last available observation.

| Categorical response | Sham | Active | p-value |
| --- | --- | --- | --- |
| Partial response (≥25%) | 6 (66%) | 10 (100%) | p=0.08 |
| Response (≥50%) | 4 (44%) | 7 (70%) | p=0.36 |
| Remission (≤9) | 2 (22%) | 5 (50%) | P=0.34 |

**Supplementary Table 3.** Correlation between MADRS change, baseline BMI and pre/post session baseline to endpoint blood pressure change.

|  |  | All patients | | Sham group | |  | Active group | |
| --- | --- | --- | --- | --- | --- | --- | --- | --- |
|  |  | r | p-value | r | p-value |  | r | p-value |
| BL BMI vs. change in MADRS |  | 0.11 | 0.665 | -0.11 | 0.781 |  | 0.24 | 0.502 |
| BL to endpoint change in systolic BP session change* vs. change in MADRS |  | 0.54 | 0.016 | 0.47 | 0.199 |  | 0.52 | 0.121 |
| BL to endpoint change in diastolic BP session change* vs. change in MADRS |  | 0.45 | 0.056 | 0.44 | 0.239 |  | 0.26 | 0.460 |
| *(BP,post session - BP,pre session)_endpoint_ - (BP,post session - BP,pre session)_baseline_ | | | | | | | | |

**Supplementary Table 4.** Exploratory metabolic parameters.

|  | **Sham** | **Active** | **p-value** |
| --- | --- | --- | --- |
| Adiponectin | 1472 (2409) | -805 (1640) | 0.444 |
| FGF-21 | -30.9 (78.1) | -13.3 (53.2) | 0.854 |
| Leptin | 2.7 (6.4) | -1.5 (4.4) | 0.593 |
| LCn-3 (EPA+DHA) | -0.01 (0.22) | -0.02 (0.19) | 0.983 |
| Insulin | -7.2 (6.8) | -4.2 (4.6) | 0.722 |
| Cortisol | -0.9 (3.1) | -0.7 (2.2) | 0.958 |

Repeated measures ANOVA considering all available data with mean (SE) shown. Abbreviations: fibroblast growth factor-21 (FGF-21); Long chain omega-3 fatty acids (LCn-3); Erythrocite eicosapentaenoic acid + docosahexaenoic acid (EPA+DHA).

**Supplementary Case Detail 1.**

Additional details are provided on the participant assigned to active tsDCS that was lost to follow up after being referred for evaluation for an “asymptomatic decrease in heart rate” at week-4 and the rationale to document this as an adverse event (AE). Briefly, the participant is an athletic young female (BMI:19.2 kg/mt2) that could run up to 6 miles per day several times per week and had decreased the amount of physical activity during her MDD episode. At the screening visit, she had a heart rate (HR) of 51 BPM and 59 BPM at baseline. Her baseline asymptomatic bradycardia was considered as non-clinically significant and reflective of her excellent physical condition.

During her week-4 study visit, the participant was showing an improvement in MADRS of 10 points from baseline and had gradually resumed running between 6 and 10 miles per day several days per week. She reported to have run 10 miles the day before. There was no evidence of manic/hypomanic symptoms. An asymptomatic HR of 47 BPM was detected during the assessment prior to the tsDCS session. Out of an abundance of caution, the tsDCS session was not conducted on that visit and she was referred for evaluation. The research team was in contact with the patient until she was evaluated by her physician five days later. At that time, her physician informed the research team that the participant had been examined and that her physical evaluation was normal and was considered to show bradycardia in an athletic person. The physician also indicated that she should not participate in any research study until such participation was cleared by Cardiology. The research team then attempted to contact the participant for follow up on multiple occasions for more than a month without success, and she was considered as lost to follow up.

As documented, the asymptomatic bradycardia was present before the intervention. At week 4, the HR decreased by a total of 4 BPM (from screen) or 12 BPM (from baseline). It remains unclear whether this is truly an adverse event considering that she is an athletic young person, that was asymptomatic, and had resumed running up to 10 miles per day several days per week during study participation. However, considering how little is known about the potential cardiovascular effects of tsDCS, we considered it was necessary to document this situation. Therefore, we opted to report it as an AE “asymptomatic decrease in heart rate”.
